# Multilevel Models for the Distribution of Hosts and Symbionts

**DOI:** 10.1371/journal.pone.0165768

**Published:** 2016-11-10

**Authors:** Maxwell B. Joseph, William E. Stutz, Pieter T. J. Johnson

**Affiliations:** 1 Earth Lab, University of Colorado, Boulder, Colorado, United States of America; 2 Ecology and Evolutionary Biology, University of Colorado, Boulder, Colorado, United States of America; University of Ostrava, CZECH REPUBLIC

## Abstract

Symbiont occurrence is influenced by host occurrence and vice versa, which leads to correlations in host-symbiont distributions at multiple levels. Interactions between co-infecting symbionts within host individuals can cause correlations in the abundance of two symbiont species across individual hosts. Similarly, interactions between symbiont transmission and host population dynamics can drive correlations between symbiont and host abundance across habitat patches. If ignored, these interactions can confound estimated responses of hosts and symbionts to other factors. Here, we present a general hierarchical modeling framework for distributions of hosts and symbionts, estimating correlations in host-symbiont distributions at the among-site, within-site, among-species, and among-individual levels. We present an empirical example from a multi-host multi-parasite system involving amphibians and their micro- and macroparasites. Amphibian hosts and their parasites were correlated at multiple levels of organization. Macroparasites often co-infected individual hosts, but rarely co-infected with the amphibian chytrid fungus. Such correlations may result from interactions among parasites and hosts, joint responses to environmental factors, or sampling bias. Joint host-symbiont models account for environmental constraints and species interactions while partitioning variance and dependence in abundance at multiple levels. This framework can be adapted to a wide variety of study systems and sampling designs.

## Introduction

Symbiotic organisms—those that live with, in, or on free living hosts—play important roles in disease dynamics, food production, and host health [1, 2]. However, host-symbiont interactions complicate efforts to explain symbiont occurrence and abundance for several reasons. First, symbiont distributions depend on host distributions. In the extreme, obligate symbionts cannot exist without hosts [3]. Symbionts also influence host distributions through effects on fitness and population dynamics [4, 5]. Further complexity arises in systems with multi-host symbionts, and host individuals infected with multiple co-infecting symbionts. Symbionts occupying the same host individual can interact, such that one symbiont may directly affect the distribution of another symbiont at the individual level [6]. Useful models of symbiont occurrence and abundance should accommodate these bidirectional influences and the hierarchical nature of host-symbiont interactions [7].

Multilevel modeling provides a promising avenue to understand patterns in host and symbiont abundance at different levels of biological organization [8]. A general host-symbiont modeling framework must be multivariate: any interaction between a host and a symbiont involves at least two species. Further, useful methods should make use of observable host and symbiont data which often consist of discrete counts, but may also include binary measurements of habitat use or continuous measures of density. Continuous and discrete multivariate observations can be modeled by combining univariate distributions with multivariate linear predictors, leading to a multivariate probit for binary data, multivariate Poisson for counts, and multivariate lognormal for continuous positive observations [9–11]. Such models are increasingly being used to model distributions of free-living species while accounting for species interactions [12–15].

While ecologists often seek to estimate the effects of one species on another species, this requires strong causal assumptions when working with observational data [16]. Instead, correlations in species abundance and occurrence—potentially resulting from species interactions—can be modeled as a proxy, helping to generate hypotheses about interactions that ideally can be pursued experimentally [17]. Due to the hierarchical nature of host-symbiont interactions, these correlations can occur at multiple levels [18]. Symbionts may be correlated at the level of host individuals, positively if two symbiont species often co-infect hosts [19]. Symbionts may also be correlated at the level of host species, positively if two symbionts tend to infect the same species [20]. Hosts and symbionts might also be correlated within and among spatial locations (hereafter “sites”). While such correlations can arise through species interactions, they can also emerge from simultaneous responses to extrinsic factors or sampling bias. These alternative drivers of correlations are not guaranteed to be identifiable from observational data alone [16, 21], emphasizing the importance of methods that limit causal assumptions.

Here we expand upon existing methods to develop a hierarchical, multivariate framework for modeling host and symbiont distributions that accounts for multiple levels of correlation, level-specific covariates, and flexible likelihood specifications. We begin by outlining the general features and logic of this approach. We then present an empirical case study of amphibian hosts and their parasites, revealing correlation among species at multiple levels and demonstrating the types of insights gained in practice. We conclude by discussing limitations and potential extensions.

## Methods

We consider a landscape with discrete habitat patches (sites) containing multiple species of hosts and symbionts. At each site, replicate surveys are conducted to measure host density, and symbiont abundance is observed by sampling individual hosts. We assume each host species *h* = 1, …, *H* is present or absent at each site *i* = 1, …, *N*, with occurrence constant across surveys. If they are present, they have some non-zero site-level average density *μ*_*ih*_. The probability of occurrence *ψ*_*ih*_ and expected density within a site if present are assumed to be proportional [22]. Hosts may be present at a site but unobserved due to sampling error [23]. Conditional on occurrence, the probability of detection increases with density [24]. In other words, sites that would favor high density are commonly occupied, and abundant hosts are easier to detect than rare hosts. At site *i*, *J*_*i*_ > 1 repeat surveys are conducted, leading to the following likelihood or sampling distribution for host abundance observations:

$$\begin{array}{c} y_{ih}∼\{\begin{array}{cc} \psi_{ih}\prod_{j=1}^{J_{i}}f\left(y_{ihj}|\theta_{ihj}\right), & \sum_{j=1}^{J_{i}}y_{ihj}>0\\ \psi_{ih}\prod_{j=1}^{J_{i}}f\left(0|\theta_{ihj}\right)+1-\psi_{ih}, & \text{otherwise} \end{array} \end{array}$$ (1)

Where *y*_*ih*_ is a vector of length *J*_*i*_ with elements consisting of abundance measurements (e.g., counts) of species *h* at site *i* in each survey. This is a mixture model with components representing cases in which species *h* is present or absent from site *i* with probabilities *ψ*_*ih*_ and 1 − *ψ*_*ih*_, respectively. Further, *f*(*y*|*θ*_*ihj*_) is a probability density or mass function with parameter(s) *θ*_*ihj*_ potentially varying across sites, species, and surveys [25]. If species *h* is not observed at site *i*, then it was absent with probability 1 − *ψ*_*ih*_ or present but unobserved with probability *ψ*_*ih*_∏_*J*_*i*__*f*(0|*θ*_*ihj*_). False absences are more likely for species with low densities and those highly aggregated within sites. For simplicity we assume that detection implies species presence, but a likelihood could be specified to account for false positives [26].

We assume that the occupancy probability of species *h* at site *i* increases with the expected density *μ*_*ih*_ as follows [27]:

$$\begin{array}{c} \text{logit}\left(\psi_{ih}\right)=\gamma_{0h}+\gamma_{1h}\log\left(\mu_{ih}\right) \end{array}$$ (2)

Here, *γ*_0*h*_ is the probability of host species *h* occurring at site *i* on a logit scale when the mean density is one individual per unit area of habitat (e.g. per square meter), and *γ*_1*h*_ is a parameter that describes the scaling between expected density and the probability of occupancy, which we expect to be positive. This occupancy submodel could also include covariates such as habitat area.

Symbiont species *s* = 1, …, *S* are present or absent at each site. At site *i*, *K*_*i*_ host individuals are sampled and their infections quantified. Non-detection of symbiont *s* at site *i* can result from true absence or failure to sample an infected host, and sites that would favor high symbiont abundance are more likely to be occupied, leading to the following likelihood for symbiont abundance observations, where *y*_*is*_ is a vector of length *K*_*i*_ containing the observed infection intensities for all hosts sampled for symbiont species *s* at site *i*:

$$\begin{array}{c} y_{is}∼\{\begin{array}{cc} \psi_{is}\prod_{k=1}^{K_{i}}f\left(y_{isk}|\theta_{isk}\right), & \sum_{k=1}^{K_{i}}y_{isk}>0\\ \psi_{is}\prod_{k=1}^{K_{i}}f\left(0|\theta_{isk}\right)+1-\psi_{is}, & \text{otherwise} \end{array} \end{array}$$ (3)

Similar to the host occurrence model component, the probability of occupancy *ψ*_*is*_ is a function of the expected infection intensity across all hosts at site *i* for symbiont *s*:

$$\begin{array}{c} \text{logit}\left(\psi_{is}\right)=\gamma_{0s}+\gamma_{1s}\log\left(\mu_{is}\right) \end{array}$$ (4)

Every host and symbiont species has a site-level mean density, and these densities may be correlated e.g., if an abundant reservoir host increases infection in other hosts [28]. Species have some among-site variance in their abundances, and these variance parameters may differ across species. Species that are always at low or high abundance will have low variance, and species that are abundant in some sites, and absent from others will have higher variance. These correlation and variance parameters are used to construct a covariance matrix **Σ**_*site*_ with elements *ρ*_*mn*_*σ*_*m*_*σ*_*n*_ in the *m*^*th*^ row, *n*^*th*^ column, where *ρ*_*mn*_ is the correlation between species *m* and *n*, and *σ*_*m*_ is the among site standard deviation for species *m*. Each site has a random effect vector ***α***_*i*_ of length *H* + *S*: ***α***_*i*_ ∼ *N*_*H*+*S*_(**0**,**Σ**_*site*_), where *N*_*d*_(**0**,**Σ**) represents a multivariate normal distribution with dimension *d*, mean vector **0**, and covariance matrix **Σ**.

Within sites, hosts and symbiont density can vary among survey locations. Uniformly distributed species have low variance, and spatially aggregated species have high variance. Species are correlated within sites if they tend to be observed together in the same surveys more or less often then expected by chance, for example. We can represent these survey level correlations and variance parameters in a covariance matrix **Σ**_*survey*_, which gives rise to to $J_{tot}=\sum_{i}j_{i}$ survey level random effect vectors ***α***_*j*_, each with length *H* + *S*: ***α***_*j*_ ∼ *N*_*H*+*S*_(**0**,**Σ**_*survey*_). Random effects may be adapted to alternative sampling designs. For instance, if hosts are sampled for symbionts independently from host density surveys, then symbionts are not associated with particular surveys and the survey-level random effects may instead have dimension *H*.

Differences in overall mean abundance are represented with a host species specific random effect *α*_0*h*_ which is univariate normally distributed around a community mean, with among species variance. Together, these random effects contribute to the expected number of individual hosts of species *h* detected in a survey *j* at site *i* when the species is present, here with a log-link:

$$\begin{array}{c} \log\left(\mu_{ihj}\right)=\alpha_{0h}+\alpha_{jh}+\alpha_{ih} \end{array}$$ (5)

Depending on survey design, this expectation might include an offset that accounts for among-survey variation in sampling time intervals or area [8].

The expected density of symbionts also includes an intercept *α*_0*s*_ and elements from the site-level and survey-level random effects. However, because of the nature of host-symbiont interactions, symbionts have the potential for correlation at additional levels. Specifically, symbionts may be correlated at the individual host level, e.g., if two symbionts commonly co-infect host individuals. We represent these host individual differences with *K*_*tot*_ = ∑_*i*_*K*_*i*_ multivariate normal random effects with mean zero and covariance matrix **Σ**_*indiv*_ including correlation terms and symbiont species specific variance terms representing how variable host individuals are in their infection abundances: ***α***_*k*_ ∼ *N*_*S*_(**0**,**Σ**_*indiv*_).

Finally, hosts may vary in their symbiont infection abundances at the species level. This variation may be correlated if two host species are functionally alike, e.g., they tend to be similarly susceptible to infection across a range of symbiont species. To allow for species level variation we consider *h* = 1, …, *H* multivariate normal random vectors, each with *S* elements: ***α***_*h*_ ∼ *N*_*S*_(**0**,**Σ**_*species*_).

Together, these random effects contribute to the expected infection load of symbiont *s* present at site *i* in host individual *k* of species *h* sampled in survey *j*:

$$\begin{array}{c} \log\left(\mu_{isk}\right)=\alpha_{0s}+\alpha_{is}+\alpha_{j[k]s}+\alpha_{h_{k}s}+\alpha_{ks} \end{array}$$ (6)

If host sampling for symbionts occurs separately from host abundance surveys, then sampled hosts are not associated with surveys, simplifying the random effects:

$$\begin{array}{c} \log\left(\mu_{isk}\right)=\alpha_{0s}+\alpha_{is}+\alpha_{h_{k}s}+\alpha_{ks} \end{array}$$ (7)

### Case study: amphibian communities and their parasites

Amphibians in the San Francisco Bay Area of California are infected with a diverse suite of parasites, including macroparasitic helminth worms (*Ribeiroia ondatrae* Looss, 1907, *Echinostoma* sp., *Cephalogonimus* sp., *Alaria* sp.), and microparasites such as *Ranavirus* sp. and the amphibian chytrid fungus *Batrachochytrium dendrobatidis*, Longcore, Pessier & D.K. Nichols (1999), hereafter referred to as *Bd*.

Five amphibian hosts comprise the majority of non-threatened (available for sampling) amphibian species: the Pacific chorus frog *Pseudacris regilla* (Baird & Girard, 1852), California newt *Taricha torosa* (Rathke, in Eschscholtz, 1833), rough-skinned newt *Taricha granulosa* (Skilton, 1849), western toad *Anaxyrus boreas* (Baird & Girard, 1852), and the non-native American bullfrog *Lithobates catesbeianus* (Shaw, 1802) [29]. Previous studies in this system have revealed correlations between parasites at the host individual and site levels [30, 31].

In 2013, field crews visited 87 wetland sites in Contra Costa, Alameda, and Santa Clara counties. At each site, crews conducted dip net sweep surveys ($\bar{J_{i}}=10.5$, standard deviation (*s*_*J*_*i*__) = 2.65, range = [2, 20], *J*_*tot*_ = 914) to quantify amphibian density, recording the numbers and species identities of all amphibians observed. Crews collected hosts at each site to quantify parasite infections ($\bar{K_{i}}=17.8$, *s*_*K*_*i*__ = 12.7, range = [1, 82], *K*_*tot*_ = 1550), and these collection events were separate from the sweep surveys. Collected hosts were larval or recently metamorphosed. We assessed macroparasite infection abundance via dissection [29] following euthanasia via immersion in MS-222 (1g/500 mL dose), and infection loads of Bd and *Ranavirus* using quantitative polymerase chain reaction of skin swabs and organ tissue [32, 33]. This work was approved by the University of Colorado Boulder IACUC, protocol number 1302.02. Access to the study sites and organisms was permitted by the California Department of Fish and Game, the Santa Clara County Parks and Recreation Department, the East Bay Regional Park District, the State of California Department of Parks and Recreation, and the East Bay Municipal Utility District.

### Prior distributions

Our prior distributions were chosen to be vague but within reasonable values given the link functions used (logit for occurrence probabilities, and log for expected abundance). Random effect covariance matrices (**Σ**_site_, **Σ**_survey_, **Σ**_indiv_, **Σ**_species_) received prior distributions specified in terms of correlation matrices and a vector of standard deviations. For example at the site level, **Σ**_site_ = diag(**σ**_site_)***R***_site_ diag(**σ**_site_)), where diag(**σ**_site_) is a diagonal matrix with a vector of species specific standard deviations that represents the amount of variation in abundance among sites for each species, and ***R***_site_ is a correlation matrix that represents correlation among species abundance among sites.

At each level for the random effects (site, survey, individual, and species), we specified log normal prior distributions for the species specific standard deviation vectors with hyperparameters to allow for partial pooling, e.g., *σ*_site,*i*_ ∼ log-Normal(*μ*_*σ*_site__, *τ*_*σ*_site__) independently for each species *i* = 1, 2, …, *H* + *S*, where the hyperparameters *μ*_*σ*_site__ and *σ*_*τ*_ represent the average among-site standard deviation across species, and the standard deviation among species in the among site standard deviations. Biologically, this allows for species to be more or less variable in abundance among sites, rather than assuming that the variability among sites is the same for all species. We assumed that *σ*_*τ*_ applied at the site, survey, individual, and species levels, but allowed the hyperparameter mean to vary at each of these levels, implying that there may be more or less variation in abundance at these levels, but that the among-species variation at each level would be constant. Future applications of this method may benefit from a more flexible specification that allows for *σ*_*τ*_ to vary among model levels, particularly if there are many species, and thus more information about this hyperparameter.

Hyperparameter priors for the means (*μ*_*σ*_site__, *μ*_*σ*_survey__, *μ*_*σ*_individual__, *μ*_*σ*_species__) and standard deviations (*σ*_*τ*_) of these log-Normal priors were specified as unit Normal and unit half-Normal (Normal_+_(0, 1)), respectively. Random effect correlation matrices (***R***_site_, ***R***_survey_, ***R***_individual_, ***R***_species_) received LKJ(*η* = 2) prior distributions which place slightly more prior weight around correlations near 0 [34]. Last, the hyperparameters for the species-specific intercepts received the following priors: *μ*_*α*_ ∼ Normal(0, 1), *σ*_*α*_ ∼ Normal_+_(0, 1). These prior specifications can be readily changed by modifying mod.stan in S1 Code.

### Parameter estimation

We used a Bayesian approach to estimate parameters, combining prior information with a Poisson likelihood to generate a posterior distribution for unknown quantities. We simulated samples from the posterior using Markov chain Monte Carlo (MCMC) sampling in the probabilistic programming language Stan [35, 36]. We ran four chains with the No-U-Turn Sampler for 1000 iterations each, discarding the first 500 iterations as burn-in [37]. Convergence was assessed visually and by verifying that all of the $\hat{R}$ statistics were less than 1.1 [38]. All data and code required to reproduce the analysis are available in S1 Code.

## Results

**Fig 1:**
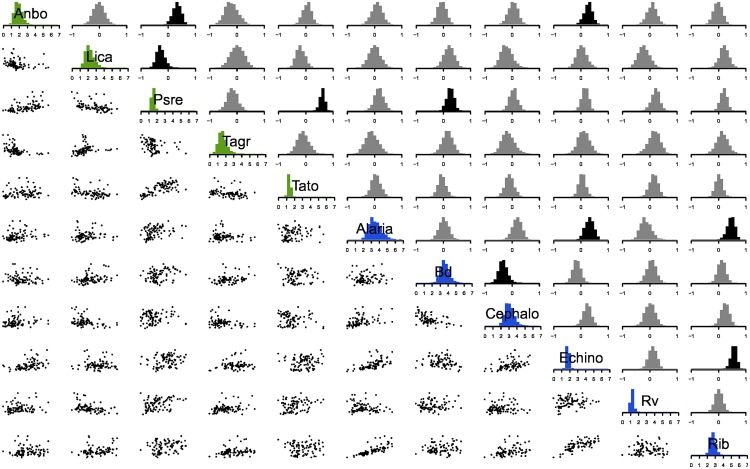
Site level variance covariance matrix and random effect posteriors. Diagonal elements display the among-site standard deviation in abundance for all host and parasite species (Anbo = *Anaxyrus boreas*, Psre = *Pseudacris regilla*, Lica = *Lithobates catesbeianus*, Tagr = *Taricha granulosa*, Tato = *Taricha torosa*, Rib = *Ribeiroia ondatrae*, Echino = *Echinostoma* sp., Cephalo = *Cephalogonimus* sp., Alaria = *Alaria* sp., Rv = *Ranavirus* sp., Bd = *Batrachochytrium dendrobatidis*). Green indicates hosts and blue, parasites. Upper triangular elements show among-species correlation parameters. Black indicates correlations that are probably positive or probably negative (95% of posterior probability mass greater than or less than zero); grey indicates otherwise. Lower triangular elements show bivariate scatter plots of the posterior means of the site-level random effects corresponding to the intersection of the species in the rows and columns, such that each site is represented by one point in each panel.

We uncovered correlations between hosts and parasites at every level in the model, with the exception of among-parasite species correlations at the host-species level. At the site level, we detected multiple correlations between hosts and parasites (Fig 1). Sites with high densities of Pacific chorus frogs (Psre) had high densities of California newts (Tato) and western toads (Anbo), possibly due to similar habitat requirements [39]. Sites with high densities of chorus frogs (Psre) had higher Bd infection loads, consistent with this species’ role as a reservoir host [40]. Sites with high levels of infection of *Cephalogonimus* (Cephalo) tended to have lower levels of infection with Bd. Macroparasites were positively correlated across sites, probably due to availability of planorbid snails that release macroparasite infective stages (cercariae), and deposition of parasite eggs in feces of carnivorous definitive hosts.

**Fig 2:**
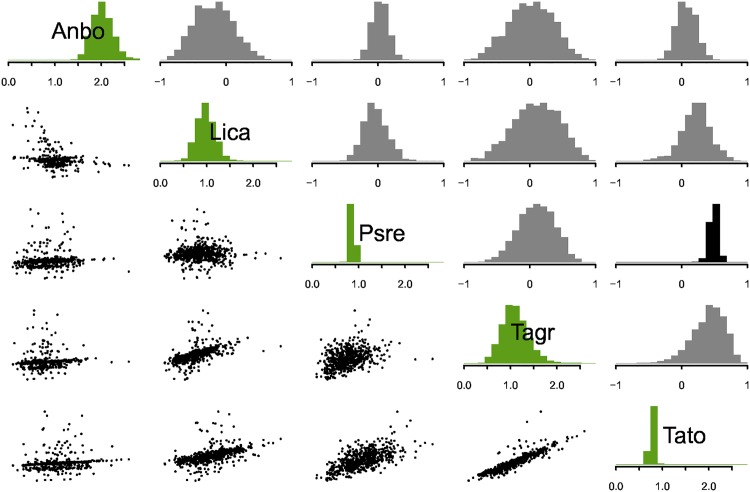
Survey level variance covariance matrix and random effect posteriors. Diagonal elements display the among-survey standard deviation in abundance for host species. Upper triangular elements show among-species correlation parameters. Black indicates correlations that are probably positive or probably negative (95% of posterior probability mass greater than or less than zero); grey indicates otherwise. Lower triangular elements show bivariate scatter plots of the posterior means of the survey-level random effects corresponding to the intersection of the species in the rows and columns, such that each survey is represented by one point in each panel.

Within sites at the survey level, California newts (Tato) correlated positively with Pacific chorus frogs (Psre) (Fig 2). These correlations imply that these species tend to be co-aggregated within sites, potentially due to similar microhabitat preferences.

**Fig 3:**
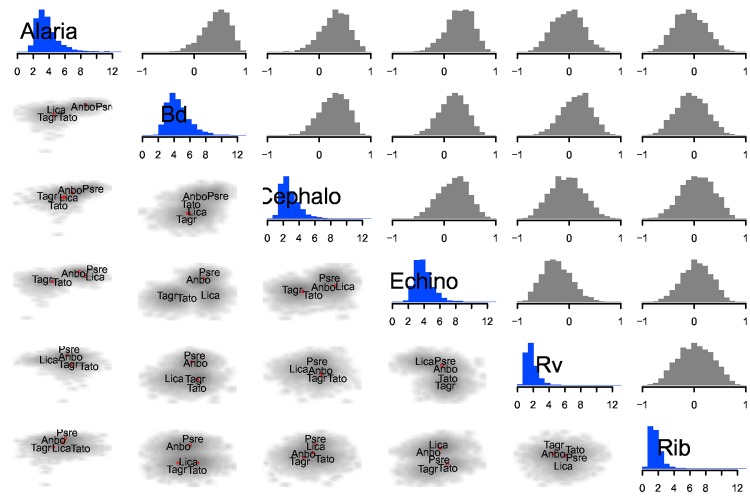
Host species level variance covariance matrix and random effect posteriors. Diagonal elements display the among host species standard deviation in abundance for parasite species. Upper triangular elements show among-species correlation parameters. Black indicates correlations that are probably positive or probably negative (95% of posterior probability mass greater than or less than zero); grey indicates otherwise. Lower triangular elements show bivariate smoothed scatter plots of species-level random effects, with host species codes printed at the posterior means. The smoothed grey portions represent the posterior densities of the species-level random effects.

At the host species level, among-parasite correlations were estimated with low precision as we would expect when trying to estimate a correlation with five points (host species). However, some posteriors leaned toward positive correlations e.g., between Bd and *Alaria* (Fig 3). This was driven by high infection abundances of most parasites in Pacific chorus frogs (Psre), consistent with these fast-lived hosts investing little in parasite defense [20]. More host species are needed to make reliable inference at this level.

**Fig 4:**
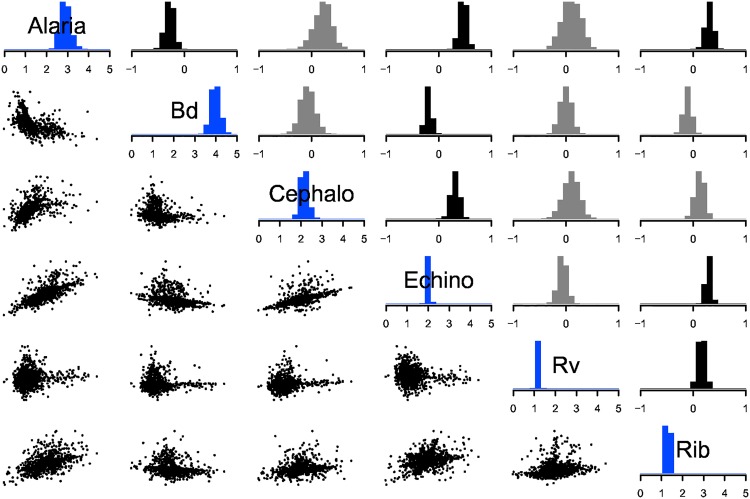
Individual level variance covariance matrix and random effect posteriors. Diagonal elements display the among host individual standard deviation in abundance for parasite species. Upper triangular elements show among-individual correlation parameters. Black indicates correlations that are probably positive or probably negative (95% of posterior probability mass greater than or less than zero); grey indicates otherwise. Lower triangular elements show bivariate scatter plots of the posterior means of the individual-level random effects corresponding to the intersection of the species in the rows and columns, such that each host individual is represented by one point in each panel.

At the individual host level macroparasite loads correlated positively, so that if an individual was heavily infected with one macroparasite, it was more likely to be heavily infected with other macroparasites (Fig 4). These positive correlations can occur despite negative within-host interactions [41]. For instance *Ribeiroia* (Rib) and *Echinostoma* (Echino) both have negative effects on the persistence of one another within host individuals, and the positive correlation may result from these parasites having similar niche requirements and host preferences [30]. In contrast, Bd correlated negatively with two macroparasites, *Alaria*, and *Echinostoma* (Echino). Parasite interactions could drive these correlations or they could result from confounding variables. For example, host age increases cumulative exposure, confounding inference on parasite interactions derived from correlations. Such correlations may disappear after including the confounding trait as a covariate, contingent on the validity of the model with respect to the true latent processes [16]. Last, correlations could arise from sampling bias [42]. For instance, if Bd or *Echinostoma* (Echino) infection increases catchability, then these two parasites will correlate negatively in our sample even if they are not correlated within the population.

**Fig 5:**
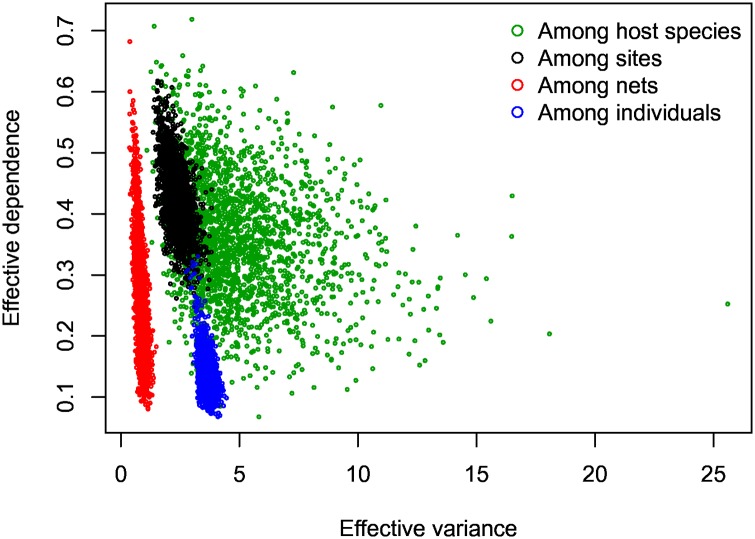
Bivariate posterior distributions of the effective variance and dependence for the multivariate random effects. Each point represents a simulated draw from the posterior. Effective variance measures the magnitude of spread in any direction of the random effects, and effective dependence measures the magnitude of among-species correlation.

We partitioned variation in host and parasite abundance among model levels to better understand the relative strength of processes operating at different scales. This analysis aims to summarize the correlations and extra-Poisson variance induced by the random effects. We considered effective variance *V*_*e*_(*X*): = |**Σ**_*X*_|^1/*d*^, the *d*–th root of the determinant of a covariance matrix **Σ**_*X*_ with dimension *d*, which represents the average scatter in any direction [43]. We also considered effective dependence *D*_*e*_(*X*): = 1 − |***R***_*X*_|^1/*d*^, where ***R***_*X*_ is a correlation matrix, which captures the stochastic dependence among species [43]. If species tend to be highly correlated, this parameter will be close to one. With no correlation among species, effective dependence is zero. Within-site, among-survey variation accounted for less variation in host abundance than among site random effects (Fig 5). For parasites, variation among host individuals exceeded among-site variation. This is striking, but consistent with the notion that parasites are overdispersed and aggregated among host individuals [44]. Despite high variance, parasite abundance showed relatively low dependence at the individual level. Effective dependence was comparable across other model levels, which might be expected if species interactions and/or joint responses to covariates similarly influence patterns of co-aggregation at these levels.

## Discussion

We presented a general hierarchical modeling framework to understand correlations and drivers of host and symbiont abundance. This builds upon existing multi-species abundance models and specifically extends a two symbiont abundance model by Stutz et al. *in review*, allowing for more than two species of symbionts, inclusion of hosts (any number of species), partially observed occurrence states, and greater flexibility in likelihood specification. Many host-symbiont distributions could be investigated with this method beyond host-parasite associations, including commensal and mutualistic symbionts of plants and animals.

This approach has been described primarily from a causally agnostic perspective, in which we are estimating unstructured correlations among species, but alternative approaches could be taken. If there is a known causal direction, e.g., in an experimental setting, one could extend this method to model the effect of host density on symbiont abundance rather than their correlation. Estimation of many covariance matrices is a rather data-hungry operation, particularly when the correlation parameters are free to vary independently. If less information were available, it may be advantageous to either include structure for the correlation parameters (e.g., [21]), or adopt a latent factor approach as recently described by [15]. In the context of host-symbiont models, latent factors could be used at multiple levels to account for unobserved site, host, and species level characteristics.

Another advantage of this joint modeling approach is the ability to decompose variation and dependence across multiple levels of organization. Effective variance and dependence may reflect the relative importance of processes at different levels of organization. For instance, we found variation among host species in parasite abundance comparable to variation among spatial locations, both of which exceeded variation within sites. Generally, the contribution of model levels to effective variance will differ among study systems, and the ability to compare across levels should be valuable in determining how to begin model expansion. In our case study for instance, a logical next step would be inclusion of site and host individual level covariates.

Alternative likelihood functions, including those accounting for measurement error, can be readily combined with this method. Here we made use of a Poisson likelihood, but some situations may call for the use of zero-inflated probability distributions with support for all real positive values, such as a zero-inflated lognormal or gamma [45]. This would allow for direct modeling of observations generated via quantitative polymerase chain reaction, typical of applications to viruses and bacteria, and environmental DNA of free-living species. Continuous distributions would circumvent the need to round values for use with Poisson or negative binomial distributions with integer support. Last, we have assumed that infections are detected without error, but a rich set of methods could be applied to account for error in this measurement process [45, 46].

We assumed that sites favoring high density are more likely to be occupied. However, if different processes drive species occurrence and abundance, then alternative occurrence submodels could be developed. In particular, spatial and temporal dependence may be useful for representing limits to species occurrence [47]. Future developments of this approach might prioritize inclusion of spatiotemporally explicit colonization dynamics that account for occupancy status of neighboring sites, habitat quality, and dispersal functions [48]. These approaches will prove useful to understand how much of the spread of an invasive symbiont may be due to changes in the host distribution vs. changes in the symbiont distribution alone, with potential applications to the management of emerging infectious diseases [49].

Symbionts have received an increased appreciation over past decades as the field of disease ecology has gained momentum and as modern genetic methods have increased our ability to sample unculturable communities [50, 51]. However, the development of methods to understand the distribution of symbionts has not kept pace with developments in free living species [52]. The approach presented here draws upon these developments with the goal of producing a general approach that can be readily adapted to other host-symbiont systems. Simultaneously modeling hosts and their symbionts in this hierarchical framework provides a powerful method to dissect patterns of occurrence and abundance for free living and symbiotic organisms.

## Supporting Information

S1 Code This supplement includes all code and data required to replicate our analysis. The analysis.R file will read and process the data from host_data.csv and parasite_data.csv, compile the Stan model (mod.stan), then estimate the parameters and recreate the figures.
(ZIP)
